# PP40 The Use Of “Softer” Outcomes In The NICE Health Technology Assessment Process: The Case Of Multiple Sclerosis

**DOI:** 10.1017/S0266462324002125

**Published:** 2025-01-07

**Authors:** Persefoni Kritikou, Alison Rose

## Abstract

**Introduction:**

Multiple sclerosis (MS) is characterized by a significant humanistic burden, as this is a chronic progressive disease associated with high levels of disability and need for caregiver support. Our objective was to explore to what extent “softer” outcome measures are considered as part of the health technology assessment (HTA) process of medications evaluated for the management of MS.

**Methods:**

We performed a review of all Technology Appraisal Guidance (TAs) available at the National Institute for Health and Care Excellence (NICE) website, relating to the management of MS, in order to identify outcomes used either by the manufacturer or discussed by the Committee, that were beyond the typical ones around the clinical evidence, side effects, and costs associated with each technology under evaluation. The review focused on outcomes relating to the caregiver burden, the ease of use of the medication under evaluation, and the fatigue reported by patients.

**Results:**

Fifteen TAs were identified. Eight (53%) of the TAs included information on the caregiver burden, where the manufacturers applied caregiver disutilities in the cost-effectiveness analyses, which were deemed appropriate by the Committee. Six (40%) of the TAs further included a discussion on the ease of use for specific medications where the Committee concluded that the benefits of the mode of administration may not have been captured in the cost-effectiveness analysis. Fatigue was discussed in two TAs; the Committee provided conflicting conclusions around this measure, but in the most recent TA, it recognized that fatigue was an important outcome measure.

**Conclusions:**

Based on the above findings, it appears that “softer” outcome measures are deemed relevant in the HTA process for treatments aimed for MS, on top of the measures typically used in the HTA process; this highlights the importance of recording the humanistic burden of disease in a holistic way.

